# Toward the Application of Dual-Energy Computed Tomography with Virtual Non-Hydroxyapatite Color-Coded Maps to Identify Traumatic Fractures in Daily Emergency Settings

**DOI:** 10.3390/jimaging10110267

**Published:** 2024-10-23

**Authors:** Claudio Ventura, Laura Maria Cacioppa, Sonia Caldarelli, Giovanni Sallei, Federico Lamponi, Marco Mascitti, Marina Carotti, Chiara Floridi, Gianluca Valeri

**Affiliations:** 1 Diagnostic Radiology Unit, Department of Services, AST Fermo, Via Augusto Murri 21, 63900 Fermo, Italy; claudio.ventura@sanita.marche.it (C.V.); sonia.caldarelli@sanita.marche.it (S.C.); gianluca.valeri@sanita.marche.it (G.V.); 2 Division of Interventional Radiology, Department of Radiological Sciences, University Politecnica Delle Marche, 60126 Ancona, Italy; c.floridi@staff.univpm.it; 3 Department of Clinical, Special and Dental Sciences, University Politecnica Delle Marche, 60126 Ancona, Italy; 4 Ortopedics Unit, Surgical Department, AST Fermo, Via Augusto Murri 21, 63900 Fermo, Italy; giovanni.sallei@sanita.marche.it (G.S.); federico.lamponi@sanita.marche.it (F.L.); 5 Division of Radiology, Department of Radiological Sciences, University Hospital “Azienda Ospedaliero Universitaria Delle Marche”, 60126 Ancona, Italy

**Keywords:** dual-energy computed tomography (DECT), material decomposition, bone marrow edema, traumatic fractures, emergency radiology

## Abstract

To evaluate the advantages of dual-energy computed tomography (DECT) virtual non-hydroxyapatite color mapping (VNHAP) in combination with standard bone CT (BCT) in the identification of subtle or occult traumatic fractures referred to emergency and acceptance departments (DEAs). Forty patients (22 men; mean age 83 ± 23.7 y) with suspected traumatic fractures referred to our emergency department and examined with a fast kilovoltage-switching single-source spectral CT scan between January and October 2023 were retrospectively reviewed. The BCT and VNHAP images were blindly evaluated by two radiologists with >10 years and <2 years of experience in musculoskeletal imaging. Both techniques were evaluated in terms of sensitivity (SE), specificity (SP), positive and negative predictive values (PPVs and NPVs) and accuracy for fracture detection, as confirmed at a 3-month clinical–instrumental follow-up. Inter-observer agreement and examination times were also analyzed. Fractures were confirmed in 18/40 cases. The highest values of diagnostic performance for VNHAP images were obtained in terms of SP (90.9% and 95%) and PPV (87.5% and 92.8%) and for the less experienced operator. No statistically significant differences were observed between the diagnostic accuracy of the two readers in the evaluation of VNHAP images. Inter-observer agreement was moderate (κ = 0.536) for BCT and substantial (κ = 0.680) for VNHAP. Comparing the two operators, a significantly longer examination time for BCT and no significant difference for VNHAP were registered. Our preliminary experience may encourage the employment of VNHAP maps in combination with BCT images in emergency settings. Their use could be time-saving and valuable in terms of diagnostic performance, especially for less experienced operators.

## 1. Introduction

Traumatic fractures are a common age-independent clinical issue and represent a major drain on the healthcare resources of all countries, due to their high morbidity, disability, and hospitalization rates [1]. In this regard, their delayed and inaccurate diagnoses negatively affect proper functional recovery over the longer term, causing additional risks and avoidable costs [2,3]. The incidence of traumatic fractures is extremely variable in different countries, based on different socioeconomic conditions. Furthermore, in many studies, fractures are overestimated due to overdiagnosis from inexperienced operators and the use of trauma center general data [4]. Another problem lies in the fact that, in larger cities, orthopedic trauma is managed in differently equipped hospitals, and data analysis is therefore biased. Despite the real incidence of traumatic fractures being difficult to establish, the evaluation of operators’ abilities in the identification of subtle or occult fractures remains an interesting subject of study, as well as the increasing use of advanced diagnostic methods to obtain precise diagnoses in a sufficiently timely manner. An accurate and effective three-dimensional imaging of traumatic fractures such as bone CT (BCT) is therefore needed, when clinical and radiographic examinations are inconclusive [4,5]. In selected cases, Magnetic Resonance Imaging (MRI), due to its high potential in detecting bone marrow edema (BME), would be extremely useful in the assessment of nondisplaced fractures. Nevertheless, it is known how logistically challenging it is to employ MRI in emergency settings [6,7,8]. First of all, MRI requires an examination time that is too long for it to be routinely performed in all clinical settings. Furthermore, the patient should lie still during examination, and this is frequently impossible for children, the elderly, or trauma patients. Recent developments have allowed for the ever-increasing dissemination of dual energy computed tomography (DECT), which enables the differentiation of materials according to their chemical composition, thus obtaining precise information on tissues’ compounds through spectral acquisition [9,10]. In the last decade, DECT, or spectral imaging, has become a decisive diagnostic method in many clinical applications, such as in neuroradiology, and cardiovascular, chest, abdominal and musculoskeletal fields [11].

Six main DECT technologies are currently available, listed as follows: (1) dual-source DECT (Siemens Healthineers, Forchheim, Germany), (2) fast kilovolt peak (kVp) switching (GE Healthcare, Milwaukee, Wisconsin), (3) dual-layer DECT (Philips Healthcare, Best, Netherlands), (4) split-filter DECT (Siemens Healthineers, Forchheim, Germany), (5) consecutive acquisitions DECT (Canon Medical Systems, Ōtawara, Japan), (6) photon-counting CT (Naeotom Alpha, Sie-mens Healthineers, Forchheim, Germany). Though all DECT technologies produce images with similar characteristics, the nomenclature varies depending on the technologies and manufacturers. One of the most recent DECT technologies is kV rapid switching, whose single x-ray source can modify voltage during a single rotation. Specific post-processing algorithms may be applied to the acquired data set for different purposes, such as subtracting a certain material to evaluate another without superposition, providing color-coded maps of tissue iodine content, and analyzing the composition of specific targets [10,11,12].This includes virtual non-hydroxyapatite application (VNHAP), which enables calcium removal and a color-coded visualization of the areas of increased bone marrow attenuation [13,14].

BME is an indirect sign of bone contusion secondary to the disruption of marrow trabeculae, with the leakage of blood and interstitial fluid to marrow spaces. The presence of BME, identified as an increased bone intensity at MRI, is often associated with acute bone pain and joint function loss and should lead to additional and careful objective examination of the traumatic sites [8,11]. The detection of BME could therefore be a useful tool to prevent adverse patient outcomes, prolonged recovery times, and avoidable complications such as dislocation, subchondral bone necrosis, osteoarthritis, and osteomyelitis. Despite the role of DECT in detecting and characterizing BME in various anatomical locations having been widely recognized, only a few recent studies support DECT as an alternative imaging method for BME in trauma patients and its effectiveness in the identification of BME in traumatic fractures [10,15,16]. In this scenario, the potential of DECT lies in a better identification of the lesion, performing a all-inclusive examination [16,17]. The aim of our study was to evaluate the value of DECT, with VNHAP as an aid to standard bone CT, in the identification of subtle or occult fractures in emergency settings.

## 2. Materials and Methods

### 2.1. Study Population and Study Design

This single-center study was approved by our Internal Review Board (IRB) and conducted in conformity to the ethics guidelines of the 1964 Declaration of Helsinki and its amendments. Due to its retrospective design, an informed consent to study participation was waived. A total of 54 patients submitted to DECT for suspected traumatic fracture in our Emergency Department between January and October 2023 were selected and retrospectively reviewed. All the considered fractures were radiographically occult or radiographically subtle. In both cases, the clinical suspicion of osseous injury with a negative or uncertain radiographic diagnosis has led to an advanced imaging examination.

**Figure 1:**
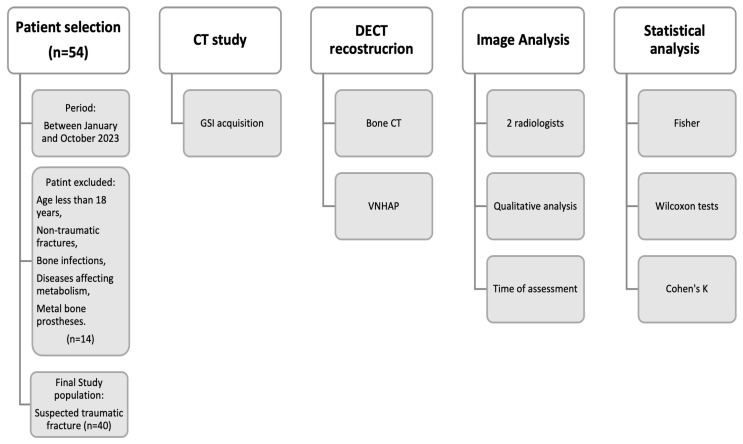
Diagram showing flowchart of patients enrolled and methodology workflow. CT = computer tomography; GSI = Gemstone Spectral Imaging; VNHAP = virtual non-hydroxyapatite.

The exclusion criteria included an age of less than 18 years, non-traumatic fractures, bone infections such as osteomyelitis, diseases affecting calcium and phosphorus metabolism, and metal bone prostheses of any type. In all the selected cases, a 3-month clinical examination, performed in the orthopedic clinic of our institution, and an X-ray or CT imaging follow-up were available. A total of 14 patients were excluded: 4 minors, 7 non-traumatic pathological fractures, 2 cases of osteomyelitis and 1 case of spondylodiscitis. The final study population included 40 patients (22 males and 18 females; mean age ± standard deviation, 83 ± 23.7 years). A flowchart the summarizing inclusion and exclusion criteria and methodology workflow is shown in Figure 1.

The diagnostic performance of BCT and VNHAP evaluated in terms of sensitivity (SE), specificity (SP), positive and negative predictive values (NPVs and PPVs), and diagnostic accuracy resulting from the analyses performed by two readers was considered as primary endpoint. The 3-month clinical evaluation and imaging confirmation obtained by radiographs, computed tomography, and/or magnetic resonance were adopted as the standard of reference.

The times taken by each reader for the evaluation of each imaging technique were considered as the secondary endpoint.

### 2.2. CT Protocol and Post-Processing

All the CT scans were obtained by a 256-slice, single-source DECT, with fast kV-switching between 80 and 140 kVp (Revolution CT, GE Healthcare^®^ Milwaukee, WI, USA). All patients were in a supine position with the head positioned towards the gantry. No intravenous contrast medium was administered. For fracture detection, axial, coronal, and sagittal reconstructions (section thickness, 0.625 mm) were reconstructed with a Bone Plus kernel. The detailed acquisition parameters were reported in Table 1. The acquired data set was postprocessed using an advanced workstation (GSI Viewer, AW Server 3.2, Ext 2.0; GE Medical Systems, Milwaukee, WI, USA). The VNHAP images were generated by two-material decomposition and displayed as color–water maps with hydroxyapatite subtraction. VNHAP maps and standard grayscale DECT series were sent to the picture archiving and communication system (PACS).

### 2.3. Image Analysis

After a training period of 20 cases, different from the study population, for the purpose of reducing any bias, the BCT images displayed on a dedicated PACS workstation (Impacs version 6.6.1.7028, Agfa Healthcare^®^, Mortsel, Belgium) were blindly and independently analyzed for the presence of fractures by two radiologists (Operator 1, SC, with >10 years of experience in musculoskeletal radiology and Operator 2, CV, with <2 years of experience). After the analysis of the BCT images, both the operators evaluated the color-coded VNHAP maps for the presence of BME. The data obtained by the two readers were compared to the 3-month clinical examinations and imaging follow-up to confirm the presence of traumatic fractures. The times taken for each individual assessment of the BCT and VNHAP images were also recorded.

### 2.4. Statistical Analysis

Continuous variables were expressed as mean ± standard deviation. The data analysis was conducted using GraphPad Prism version 9.1.1 (GraphPad Software, Boston, MA, USA) statistical software and Excel 2021^®^. Qualitative variables were analyzed by Fisher’s test. Wilcoxon’s matched-pairs signed-rank test was used for qualitative variables and for quantitative variables with abnormal distribution. The overall diagnostic performance of the visual assessment of BCT and VNHAP images was calculated according to the following formulas: true positive (TP)/true positive (TP) + false negative(FN) for sensitivity; true negative (TN)/true negative (TN) + false positive (FP) for specificity; (TP)/(TP + FP) for positive predictive value (PPV); (TN)/(TN + FN) for negative predictive value (NPV); (TP + TN)/(TP + TN + FP+ FN) for accuracy. Inter-observer agreement was measured using Cohen’s κ test and interpreted as follows: for values 0.01–0.20, the agreement was poor; for values between 0.21 and 0.40, the agreement was modest; for values between 0.41 and 0.6, the agreement was moderate; for values between 0.61 and 0.80, the agreement was substantial; and for values between 0.8 and 1, the agreement was excellent. The significance levels were set at *p* < 0.05.

## 3. Results

Over a total of 40 patients, a traumatic fracture was detected and confirmed at follow-up in 18 (45%) cases. Four (10%) vertebral fractures, four (10%) hip fractures, four (10%) wrist fractures, three (7.5%) ankle fractures, and one (2.5%) knee fracture were the most frequently detected lesions. Traumatic fractures were associated with local pain in 36 (90%) cases, local swelling in 24 (60%) cases, and determined a functional compromise in 28 (70%) cases. The demographic, etiological, and clinical characteristics of the study population were summarized in Table 2.

**Figure 2:**
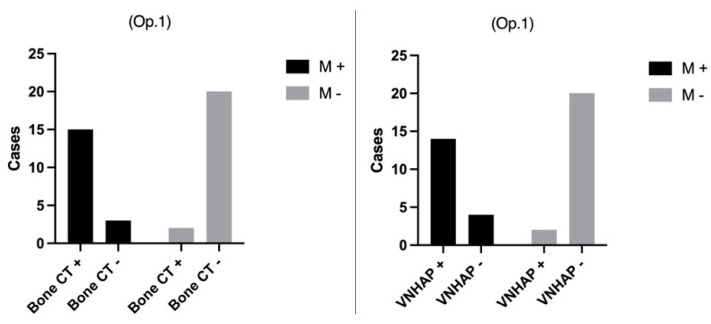
Operator 1’s evaluation of standard bone CT images and virtual non-hydroxyapatite maps (VNHAP) compared to the 3-month clinical and imaging follow-up using Fisher’s exact test. Op. 1 = Operator 1; M+ = positive for fracture; M− = negative for fracture; VNHAP = virtual non-hydroxyapatite; bone CT = bone computer tomography.

**Figure 3:**
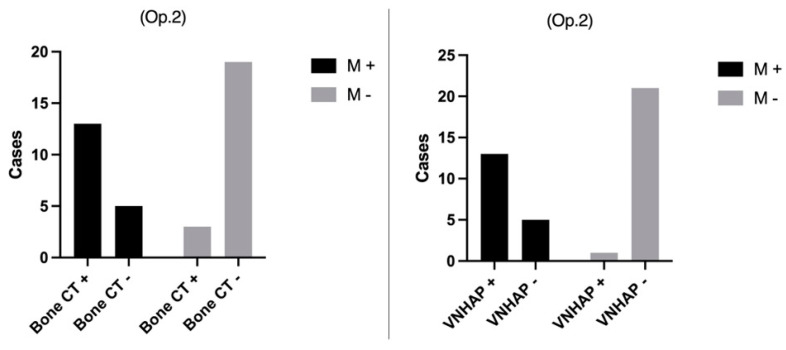
Operator 2’s evaluation of standard bone CT images and virtual non-hydroxyapatite maps (VNHAP) compared to the 3-month clinical and imaging follow-up using Fisher’s exact test. Op. 2 = Operator 2; M+ = positive for fracture; M− = negative for fracture; VNHAP = virtual non-hydroxyapatite; bone CT = bone computer tomography.

The results of visual examinations of BCT and VNHAP images performed by Operator 1 and Operator 2 were illustrated in Figure 2 and Figure 3, respectively.

The visual assessment of BCT images performed by Operator 1 and compared to a clinical–instrumental 3-month evaluation resulted in SE, SP, PPV, VPN, and accuracy values of 83.3%, 90.9%, 88.2%, 86.9% and 87.5%, respectively. Accordingly, the evaluation of BCT images performed by Operator 2 showed SE, SP, PPV, VPN, and accuracy values of 72.2%, 86%, 81.2%, 79.1% and 80.0%, respectively. Concerning the assessment of VNHAP maps, SE, SP, PPV, VPN, and accuracy values of 77.7%, 90.9%, 87.5%, 83.3%, and 85.0% for Operator 1 and SE, SP, PPV, VPN, and accuracy values of 72.2%, 95%, 92.8%, 80.7%, and 85.0% for Operator 2 were recorded, respectively. No statistically significant differences were observed between the diagnostic accuracy of the two readers in the evaluation of VNHAP images. The highest values of diagnostic performance for VNHAP images were obtained in terms of SP (90.9% and 95%) and PPV (87.5% and 92.8%) and for the less experienced operator. The cumulative diagnostic performance of the visual assessment of BCT and VNHAP images was summarized in Table 3.

**Figure 4:**
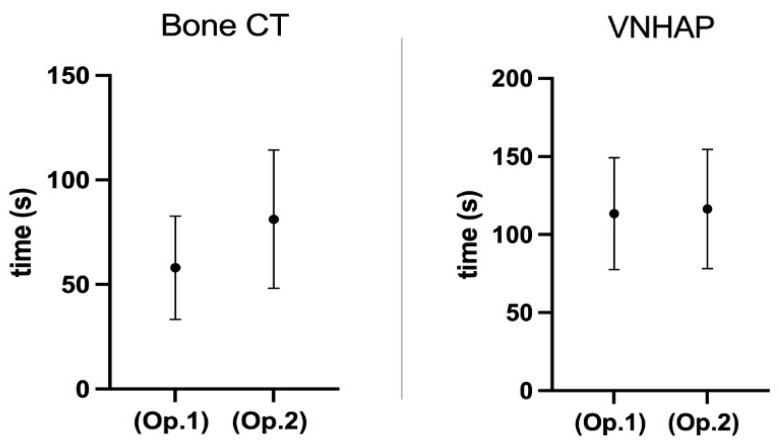
The times taken by Operator 1 and Operator 2 for bone CT evaluations (*p* < 0.0001) and VNHAP evaluations (*p* < 0.127). The results were obtained by Wilcoxon matched-pairs signed rank test. Op. 1 = Operator 1; Op. 2 = Operator 2; VNHAP = virtual non-hydroxyapatite; bone CT = bone computer tomography.

The inter-observer agreement showed a moderate agreement between the two operators when applied to BCT evaluation results (Cohen’s κ = 0.536), instead of what was obtained in the evaluation of VNHAP color maps, wherein a substantial agreement (Cohen’s κ = 0.680) had been registered. Statistically significant differences in terms of evaluation time were obtained for BCT images as follows: 58.7 ± 25 s for Operator 1 and 81.2 ± 33.1 s for Operator 2 (*p* < 0.0001), as shown in Figure 4.

Conversely, no statistically significant differences in VNHAP image evaluation time between the more experienced and the less experienced radiologists were documented (110.8 ± 39.1 s vs. 114.9 ± 41.6 s; *p* < 0.127).

## 4. Discussion

**Figure 5:**
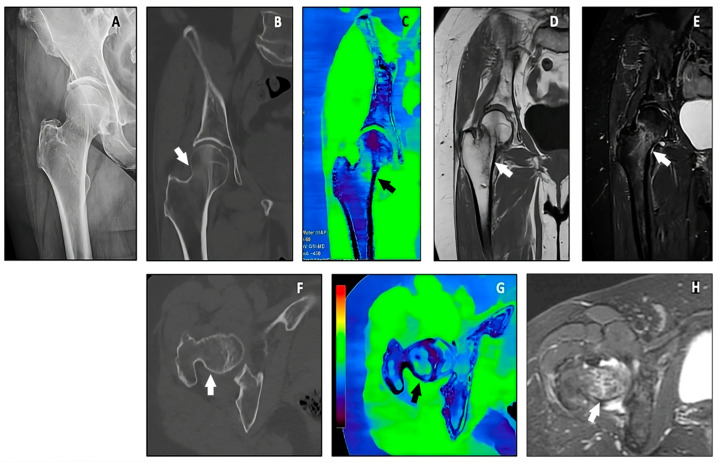
A patient referred for direct trauma to the greater trochanter with right hip pain on acupressure and during walking. A plain radiograph of the right hip showing a compound fracture of the right femoral neck (white arrows) with a transverse course (**A**), confirmed upon bone reconstruction coronal and axial CT images (**B**,**F**); 1.5 T MRI coronal T1W highlighted the fracture (**D**), and coronal and axial STIR images highlighted the bone marrow edema (**E**,**H**). VNHAP coronal and axial reconstructions confirmed the presence of bone marrow edema at the fracture site (**C**,**G**).

Since its introduction into clinical practice, DECT has achieved numerous and relevant applications in different fields such as oncological, vascular, urological, and musculoskeletal imaging [13,14,18,19,20,21]. Significant results have been obtained in the identification of metastases and primitive lesions, in perfusion studies, and in the characterization of plaques, crystals, and stones [12,18]. In musculoskeletal imaging, several interesting DECT applications have been developed and have become easily accessible with the use of specific post-processing algorithms. The principal ones in musculoskeletal imaging are represented by crystal detection in crystal-induced arthropathies, such as gout disease; the reduction of metal beam-attenuating artifacts; the characterization of soft and collagenous tissues such as tendons, ligaments and vertebral disks; the analysis of bone mineral density; and the detection of BME [11]. The study of bone marrow edema was first carried out in the clinical routine of inflammatory bone and rheumatic diseases, in malignant bone marrow infiltrations, and in chronic pain conditions such as algoneurodystrophy [11,12]. In recent studies, the detection of BME has also proven to be of extreme interest in daily urgent and emergency settings, where MRI is infrequently employed due to the long execution times, low availability, and multiple contraindications [6,22,23]. In both settings, DECT imaging may represent a potential alternative to MRI for acute traumas, especially in elderly or unstable patients. Several DECT technologies such as double-tube, sequential, dual-layer, and single-tube rapid kV switching are currently capable of identifying bone edema [24]. In previous studies, fast kV-switching DECT demonstrated similar results in terms of diagnostic performance compared to other technologies [25]. Furthermore, the HAP-water decomposition technique of rapid kV-switching DECT has previously been described as a feasible tool in the detection of abnormal edema in vertebral compressive fractures [10]. In our study, the utility of VNHAP maps in the identification of traumatic BME not identifiable with standard bone reconstruction (Figure 5) was confirmed by notable results in terms of sensitivity, specificity, and diagnostic accuracy.

The performance of the two readers in the visual analysis of BCT and VNHAP images demonstrated different results in terms of sensitivity, specificity, PPV and NPV, as expected from the different experience levels. However, no statistically significant differences were observed between the diagnostic accuracy of the two readers in the evaluation of VNHAP images. The visual analysis of VNHAP images also showed greater SP and PPV in the evaluation performed by the operator with less experience in musculoskeletal imaging.

The inter-reader agreement for VNHAP images was substantial (κ = 0.680), in concordance with previously published research, indicating that the VNHAP algorithm could be considered a promising technique for undetectable fractures on bone reconstruction CT images in an emergency workflow. In agreement with the previous series, our qualitative analysis findings depended on multiple technical factors, the personal routine of the readers, and also on the different expertise of the two readers.

Previous studies, mostly focused on vertebral fractures, have demonstrated the high diagnostic performance of DECT, by means of virtual non-calcium reconstructions, in the identification of the presence and extent of BME [15,16,26]. Compared to MRI, whose advantages stem from its precise evaluation of soft tissues and disco-ligamentous structures, DECT showed higher sensitivity and diagnostic confidence in the assessment of acute fractures, especially in cases of subtle or complex orientated fracture lines [16,27]. In this scenario, several studies in the literature have described the use of Virtual NonCalcium (VNCa) rather than VNHAP maps in the identification of traumatic bone edema, from the spine to the appendicular skeleton, demonstrating high values in terms of sensitivity and specificity [26,28,29,30]. However, the utility of single-source, fast kV-switching Gemstone™ Spectral Imaging (GSI) VNHAP maps in identifying traumatic bone edema and the employment of this technique in emergency settings and in appendicular traumatic fractures has not been reported in the literature.

**Figure 6:**
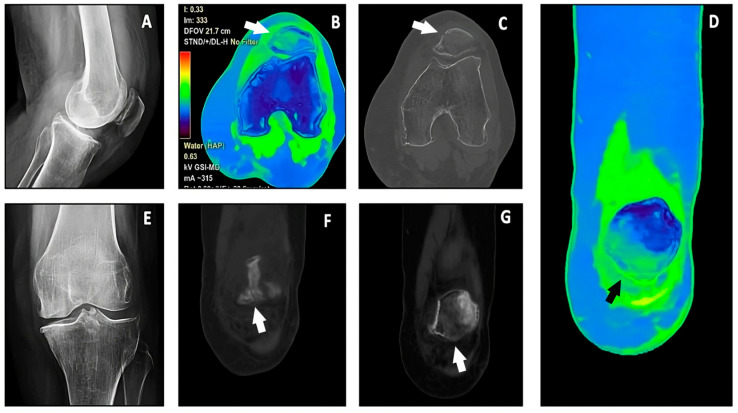
A patient referred for direct trauma to the left lateral condyle-patella, with severe pain on acupressure on the lateral condyle and functional impotence. A plain radiograph of the left knee showing a suspected compound fracture of the lower edge of the left patella (white arrows) (**A**,**E**), further confirmed by the bone reconstruction of axial (**C**) and coronal CT images (**F**,**G**). VNHAP axial and coronal color-coded maps confirmed the presence of bone marrow edema at the fracture site (**B**,**D**).

A series of 25 cases evaluated by Reddy et al. assessed the use of DECT in emergency settings for the detection of BME in hip fractures of patients with normal radiographs. This study obtained highly sensitive but poorly specific results (SE, SP, PPV, and NPV values of 90%, 40%, 86%, and 50%, respectively) compared to the present study. The difference could be related to the considered district. The reference study examined the hip district alone, where BME could be frequently due to degenerative changes rather than traumas, while in the present study different districts were investigated [6]. Furthermore, in our study, the diagnostic performance of DECT imaging was compared to a 3-month clinical examination, conducted in the same referral orthopedic department, as well as an imaging follow-up performed in the same institution for the presence of previous traumas, with the aim of matching the process as closely as possible to real daily clinical practice (Figure 6).

Several important limitations of the present study must be mentioned. First, the limitations include the limited number of patients, the retrospective design, the limited number of readers evaluating images, the lack of an MRI confirmation of BME, and the fact that pediatric cases were not taken into consideration. Secondly, many factors such as age, drugs, comorbidities, and unknown conditions modifying bone mineral density could influence DECT attenuation values and VNHAP maps. The lack of a quantitative evaluation of VNHAP maps could be considered another limitation, although a recent meta-analysis about the diagnostic performance of DECT for BME detection recommended the qualitative assessment of DECT findings rather than a quantitative assessment for a more sensitive diagnosis of BME (SE 85%; SP 97% vs. SE 84%; SP 88%) [31].

## 5. Conclusions

Regarding clinical impact, our preliminary data may encourage the use of DECT-GSI virtual non-hydroxyapatite–water decomposition color maps in the evaluation of suspected fractures in the emergency departments. The employment of VNHAP maps for the detection of BME in emergency settings was particularly valuable in terms of SP and PPV and for less experienced operators, without being time consuming.

## Figures and Tables

**Table 1:** Dual-energy computed tomography (DECT) scanning parameters.

DECT Parameters	Values
Reconstruction slice thickness (mm)	2
Reconstruction FOV * diameter (mm)	120–500
Slice Thickness (mm)	0.625
Scan-Pitch Ratio	0.516:1
Low energy (kVp)	80
High energy (kVp)	140
Tube current (mA)	190–480
Rotation Time (s)	0.8
Reconstruction algorithm	50% ASIR V **
Reconstruction kernel	Bone Plus

* FOV = Field of view; ** ASIR V = Adaptive Statistical Iterative Reconstruction.

**Table 2:** The demographic, etiological, and clinical characteristics of study population.

Demographic Features	
**Patients (n)**	40
Age (yrs) mean ± standard deviation [Range]	83 ± 23.7 [22–95]
Sex (male/female)	22/18
**Trauma history**	**n (%)**
Positive history for trauma	40 (100%)
Confirmed fracture	18 (45%)
Negative for fracture	22 (55%)
**Etiological classification**	**n (%)**
Vertebral body fractures	4 (10%)
Hip fractures	4 (10%)
Knee fractures	1 (2.5%)
Wrist fractures	4 (10%)
Ankle fractures	3 (7.5%)
Foot fractures	1 (2.5%)
Hand fractures	1 (2.5%)
**Clinical features**	**n (%)**
Local pain	36 (90%)
Local swelling	24 (60%)
Functional compromise	28 (70%)
Acute hemorrhage	0

**Table 3:** Cumulative diagnostic performance (qualitative assessment) of the visual assessment performed by Operator 1 and Operator 2 for the bone CT and virtual non-hydroxyapatite (VNHAP) images.

Variables	Operator 1		Operator 1		Operator 2		Operator 2	
	Bone CT		VNHAP		Bone CT		VNHAP	
**SE**	15/18	83.3%	14/18	77.7%	13/18	72.2%	13/18	72.2%
**SP**	20/22	90.9%	20/22	90.9%	19/22	86.0%	21/22	95.0%
**PPV**	15/17	88.2%	14/16	87.5%	13/16	81.2%	13/14	92.8%
**NPV**	20/23	86.9%	20/24	83.3%	19/24	79.1%	21/26	80.7%
**Accuracy**	35/40	87.5%	34/40	85.0%	32/40	80.0%	34/40	85.0%

SE = sensitivity; SP = specificity; NPV = negative predictive value; PPV = positive predictive value.

## Data Availability

The original data presented in the study are openly available in anonymized fashion in Google Drive at the following link: “https://docs.google.com/spreadsheets/d/15_e2gAfuTH_qYPoRaG7lh-SnuUmdp-s8/edit?usp=drive_link&ouid=111955708345406448056&rtpof=true&sd=true (accessed on 23 September 2024)”.
